# Preventing hepatitis B virus infection: milestones and targets

**DOI:** 10.2471/BLT.18.215210

**Published:** 2018-07-01

**Authors:** Yvan Hutin, Shalini Desai, Marc Bulterys

**Affiliations:** a Global Hepatitis Programme, World Health Organization, avenue Appia 20, 1211 Geneva 27, Switzerland.; b Department of Immunization, Vaccine and Biologicals, World Health Organization, Geneva, Switzerland.

Hepatitis B virus (HBV) is a major cause of mortality and morbidity. In this issue of the Bulletin of the World Health Organization, a systematic review and meta-analysis^1^ assesses the long-term impact of immunization of infants against hepatitis B on the prevalence of HBV infection. The findings from this study point to three major conclusions. First, hepatitis B vaccine is effective in preventing HBV infection among infants and has a major impact on the prevalence of HBV infection more than 15 years later. Second, universal immunization of infants, as recommended by the World Health Organization (WHO),^2^ has more impact on a population level (reducing prevalence by three fourths) than targeted immunization of children born to mothers who have HBV infection (which reduces prevalence by two thirds). Third, the results of this analysis, along with recent reports of increasing coverage of hepatitis B vaccine worldwide,^3^ suggest that in an increasing number of countries, new generations are growing up increasingly free of HBV infection.

These conclusions have important implications for the global elimination of hepatitis B.^4^ First, immunization, with one birth dose followed by two additional doses in infancy, is the foundation of efforts towards the incidence reduction target of elimination.^4^ Further work remains to be done in ensuring that a hepatitis B vaccine birth dose is added to routine immunization programmes. Only 101 countries have universal birth dose vaccination, another 20 have targeted programmes, while the public sector in the remaining countries do not administer hepatitis B vaccine to newborns. The largest proportion of infants who do not receive a birth dose is in the WHO African Region. In countries with an immunization programme in place, the long-term effectiveness of hepatitis B vaccine in the prevention of chronic HBV infection will lead to a second-generation effect of hepatitis B vaccine in the prevention of HBV transmission at birth and in young children. Children who were vaccinated 20 years ago are now becoming parents. With a lower prevalence of HBV infection in pregnant women, the vicious cycle of perinatal transmission can be broken. In addition, when fathers are immunized, decreased household or horizontal transmission means that the risk of infection to small children is also reduced. As a result, the public health benefit of hepatitis B immunization is amplified. Second, progress in the elimination of horizontal transmission of HBV among young children means that the hepatitis B prevention agenda will progressively expand towards the prevention of intrapartum and intrauterine transmission of HBV. Expanding the agenda, by including additional measures, such as hepatitis B immunoglobulins and antiviral medicine for women with high hepatitis B viral load, can further reduce mother to child transmission.^3^^,^^5^ Third, we need to work towards morbidity and mortality reduction for the 257 million persons living with HBV infection worldwide.^6^ Among these persons living with HBV, adults are at high risk of progression towards chronic liver disease, cirrhosis and hepatocellular carcinoma.^7^ Mortality, mostly through decompensated cirrhosis and hepatocellular carcinoma, almost reached 900 000 deaths in 2015, and will continue to increase if testing and treatment are not scaled up.^6^ Today, antiviral medicines are available for the treatment of HBV infection and are effective in preventing cirrhosis, hepatocellular carcinoma and death. The patents for these medicines have expired and in 2016, WHO's Global Price Reporting Mechanism GPRM reported that the median price for one year of treatment with generic tenofovir was 32.24 United States dollars.^8^

In 2016, the World Health Assembly examined challenges and opportunities and considered that elimination of HBV as a public health threat was possible.^4^ For the incidence reduction goal, the next global milestone will be to reach under 1% prevalence in children five years of age by 2020.^4^ This can be achieved through vaccination, including a birth dose, and measured through biomarker surveys that estimate the prevalence of HBV infection among children.^9^ Achievement of the 2020 target will then pave the way for the ambitious 2030 target of reducing the prevalence of HBV infection to 0.1%.^4^ WHO is defining the interventions needed and the methods that will be used to achieve and measure the 2030 target. These methods will give a greater role for the prospective follow-up of children born to HBV-infected mothers and converge with methods and approaches now used to prevent perinatal infections with human immunodeficiency virus and syphilis, with the important addition of universal immunization.^10^ This could lead to integrated triple elimination of mother-to-child transmission of all three infections.^11^^,^^12^
